# Trends in socioeconomic inequalities in preventable mortality in urban areas of 33 Spanish cities, 1996–2007 (MEDEA project)

**DOI:** 10.1186/s12939-015-0164-0

**Published:** 2015-04-01

**Authors:** Andreu Nolasco, Joaquin Moncho, Jose Antonio Quesada, Inmaculada Melchor, Pamela Pereyra-Zamora, Nayara Tamayo-Fonseca, Miguel Angel Martínez-Beneito, Oscar Zurriaga, Mónica Ballesta, Antonio Daponte, Ana Gandarillas, Mª Felicitas Domínguez-Berjón, Marc Marí-Dell’Olmo, Mercè Gotsens, Natividad Izco, Mª Concepción Moreno, Marc Sáez, Carmen Martos, Pablo Sánchez-Villegas, Carme Borrell

**Affiliations:** Unidad de Investigación de Análisis de la Mortalidad y Estadísticas Sanitarias. Departamento de Enfermería Comunitaria, Medicina Preventiva y Salud Pública e Historia de la Ciencia. Campus de San Vicente del Raspeig s/n. Apartado 99, Universidad de Alicante, 03080 Alicante, España; Registro de Mortalidad de la Comunidad Valenciana, Servicio de Estudios Epidemiológicos y Estadísticas Sanitarias, Subdirección General de Epidemiología y Vigilancia de la Salud. Conselleria de Sanitat, Plaza de España 6, 03010 Alicante, España; Área de Desigualdades en Salud. FISABIO-CSISP, Conselleria de Sanitat, Avenida de Cataluña, 21, 46020 Valencia, España; Servicio de Estudios Epidemiológicos y Estadísticas Sanitarias, Subdirección General de Epidemiología y Vigilancia de la Salud. Conselleria de Sanitat, Avenida de Cataluña, 21, 46020 Valencia, España; Ciber de Epidemiología y Salud Pública CIBERESP, Instituto de Salud Carlos III, Melchor Fernández Almagro, 3-5 28029 Madrid, España; Department of Epidemiology, Regional Health Council, Murcia, Spain; Observatorio de Salud y Medio Ambiente de Andalucía (OSMAN). Escuela Andaluza de Salud Pública, Campus Universitario de Cartuja, Cuesta del Observatorio, 4. Ap. Correos 2070, Granada, 18080 España; Servicio de Epidemiología. Subdirección de Promoción de la Salud y Prevención. Dirección General de Atención Primaria, Consejería de Sanidad Comunidad de Madrid, C/ San Martín de Porres, n° 6, 1ª planta, 28035 Madrid, España; Servicio de Informes de Salud y Estudios. Subdirección de Promoción de la Salud y Prevención. Dirección General de Atención Primaria, Consejería de Sanidad Comunidad de Madrid, C/ San Martín de Porres, n° 6, 1ª planta, 28035 Madrid, España; Agència de Salut Pública de Barcelona, Plaça Lesseps, 1, 08023 Barcelona, España; Institut d’Investigació Biomèdica (IIB Sant Pau), Barcelona, Spain; Dirección General de Salud Pública y Consumo, Gobierno de La Rioja, Calle Vara de Rey n° 8, 1ª planta, 26071 Logroño, España; Instituto de Salud Pública y Laboral de Navarra, C/ Leyre, 15, 31003 Pamplona, Navarra Spain; Grupo de Investigación en Estadística, Econometría y Salud (GRECS), [Research Group on Statistics, Econometrics and Health (GRECS)], Universidad de Girona. Calle de la Universidad 10, Campus de Montilivi, 17071 Girona, España; Instituto Aragonés de Ciencias de la Salud, Avda. San Juan Bosco, n°13, 50009 Zaragoza, España

**Keywords:** Preventable avoidable mortality, Causes of death, Inequalities in health, Small area analysis

## Abstract

**Background:**

Preventable mortality is a good indicator of possible problems to be investigated in the primary prevention chain, making it also a useful tool with which to evaluate health policies particularly public health policies. This study describes inequalities in preventable avoidable mortality in relation to socioeconomic status in small urban areas of thirty three Spanish cities, and analyses their evolution over the course of the periods 1996–2001 and 2002–2007.

**Methods:**

We analysed census tracts and all deaths occurring in the population residing in these cities from 1996 to 2007 were taken into account. The causes included in the study were lung cancer, cirrhosis, AIDS/HIV, motor vehicle traffic accidents injuries, suicide and homicide. The census tracts were classified into three groups, according their socioeconomic level. To analyse inequalities in mortality risks between the highest and lowest socioeconomic levels and over different periods, for each city and separating by sex, Poisson regression were used.

**Results:**

Preventable avoidable mortality made a significant contribution to general mortality (around 7.5%, higher among men), having decreased over time in men (12.7 in 1996–2001 and 10.9 in 2002–2007), though not so clearly among women (3.3% in 1996–2001 and 2.9% in 2002–2007). It has been observed in men that the risks of death are higher in areas of greater deprivation, and that these excesses have not modified over time. The result in women is different and differences in mortality risks by socioeconomic level could not be established in many cities.

**Conclusions:**

Preventable mortality decreased between the 1996–2001 and 2002–2007 periods, more markedly in men than in women. There were socioeconomic inequalities in mortality in most cities analysed, associating a higher risk of death with higher levels of deprivation. Inequalities have remained over the two periods analysed. This study makes it possible to identify those areas where excess preventable mortality was associated with more deprived zones. It is in these deprived zones where actions to reduce and monitor health inequalities should be put into place. Primary healthcare may play an important role in this process.

## Background

The use of avoidable mortality as a measure of the performance of healthcare services was first introduced by Rutstein [1], who presented the first theoretical study on this issue, where he proposed a list of unnecessary diseases and disabilities or unnecessary untimely deaths, based on the assertion that if health services had acted correctly, they would have been prevented or delayed. The definition and concept of avoidable mortality, as well the list of conditions considered sentinel health events, have changed over time [2-9] in line with developments in medicine and technology.

Avoidable mortality can be disaggregated into two groups [10], according to the type of healthcare intervention: 1) Preventable mortality – having to do with primary prevention, lifestyle, intervention programmes, etc. and 2) Amenable mortality – having to do with secondary prevention and directly with healthcare interventions, in the form of counselling, diagnosis or treatment.

The WHO World Health Report 2000 [11] defines health systems inclusively, as systems whose primary aim is to promote, restore and maintain health. From this point of view, preventable mortality must be considered a good indicator of possible problems to be investigated in the primary prevention chain, both in health promotion and protection and in health education [5], making it also a useful tool with which to evaluate health policies, particularly public health policies [12].

Studies conducted in several European countries have linked population socioeconomic indicators with avoidable mortality [13-17] and, in particular with preventable mortality, as a whole or in relation to specific conditions included under the definition showing higher mortality rates in the least favoured groups [18-27]. These inequalities are themselves a risk factor for population health and need to be studied in order to identify the most vulnerable groups and regions, to put in place specific interventions [28].

In recent decades, improvements in living conditions and the increasing inclusiveness of healthcare systems have reduced premature and, accordingly, avoidable mortality, both amenable and preventable. Several studies have analysed trends in mortality from avoidable causes over time in specific regions or groups [5,7,29-32] and found a decrease, although other studies described increases in avoidable mortality [33].

Some studies have associated this trend with socioeconomic inequalities, pointing to maintained and even increased socioeconomic inequalities in avoidable mortality in recent years [13,19,34-38]. Some have analysed avoidable mortality in small areas [39-41] or combined their analysis with a study of the relationship to inequality [13,15,22,27], associating the most deprived areas with higher mortality rates.

While improvements in indicators such as preventable and amenable mortality continue to be analysed to evaluate the quality, access and equity of healthcare systems [9,42-44], it is also necessary to continue to identify the zones associated with a higher risk of these causes of mortality in the urban areas of large cities, where so much of the population is concentrated, in order to take specific public health actions aimed at decreasing mortality and reducing inequalities. Studies in small areas of cities are important as neighbourhood is recognised as a health determinant independently of individual determinants [45]. In Spain no study has been conducted to date on overall preventable mortality in small areas of large cities, so the aim of this study was to describe trends in preventable mortality and analyse its relationship to socioeconomic inequalities in small areas of 33 large cities between 1996–2001 and 2002–2007.

## Methods

**Figure 1:**
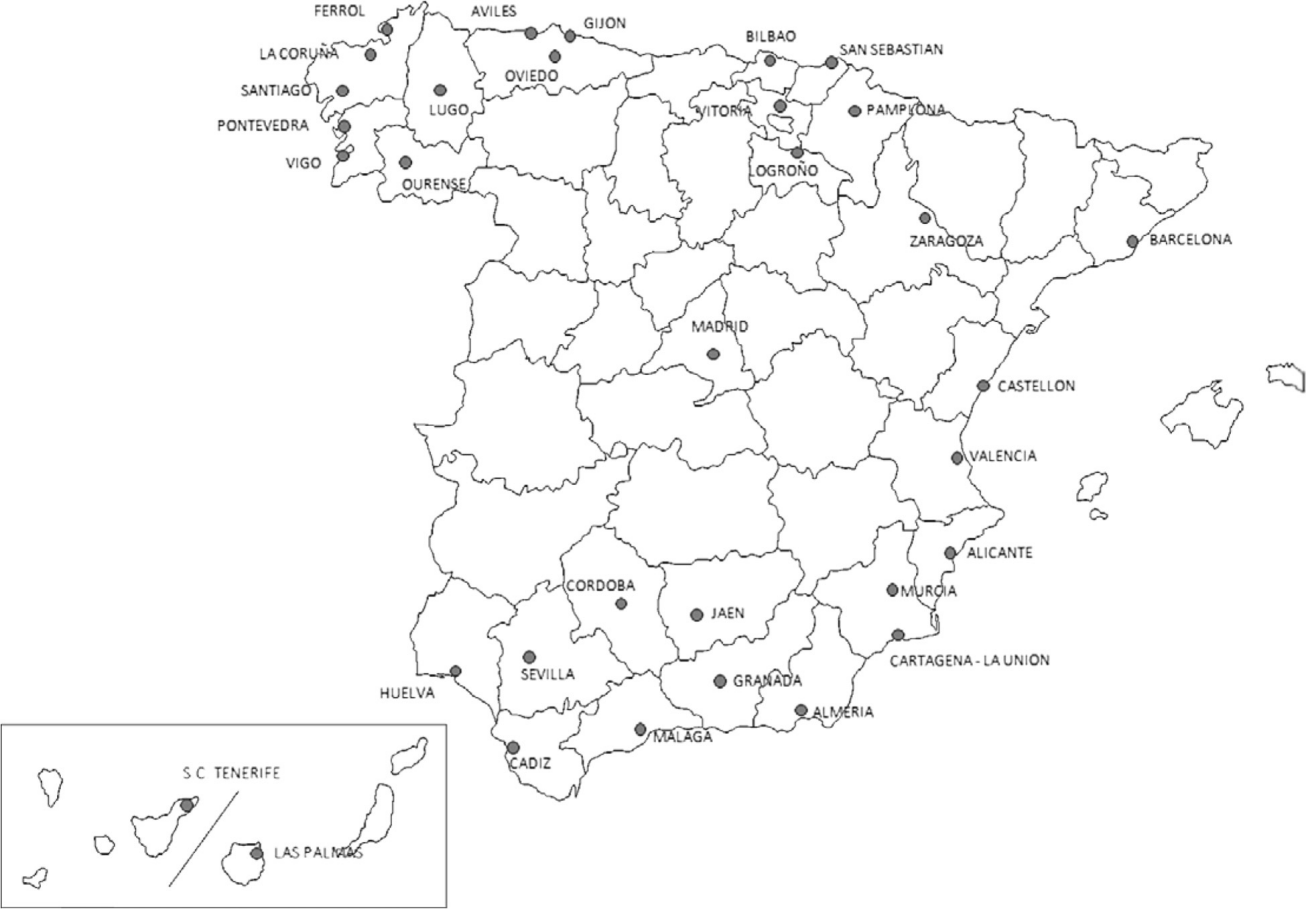
**Location of the cities analyzed.**

This study was performed within the framework of the MEDEA project (Socioeconomic and environmental inequalities in mortality in small areas of Spanish cities: http://www.proyectomedea.org) as an ecological study on preventable mortality trends in small areas of 33 Spanish cities (Figure 1) in the 1996–2001 and 2002–2007 periods. The population of these cities accounted for 30.1% of the Spanish population in 2001, according to figures from the Spanish National Statistics Institute (NSI). The units analysed were Census Tract (CT) and all deaths occurring in the population residing in these cities from 1996 to 2007 were taken into account.

The mortality figures for each CT were obtained from the death records of the corresponding autonomous community. Deaths were assigned to census sector according to their postal address. The percentage of deaths which could not be assigned to a CT due to problems in locating the residence varied from 0.02% in Pamplona to 5.0% in Cartagena-La Unión. Population figures for each CT, sex and age group (five year intervals) were obtained from the NSI. For each CT the indicators necessary for socioeconomic classification were obtained from the 2001 Population and Housing Census.

The causes of avoidable mortality included in the study were considered preventable in the MEDEA project (Table 1) and are lung cancer, cirrhosis, Acquired Immune Deficiency Syndrome and Human Immunodeficiency Virus infection (AIDS and HIV), considered together, motor vehicle traffic accidents injuries, suicide and homicide. These causes are based on those proposed by Nolte and McKee [5], adding AIDS and HIV, suicide and homicide, because of their particular importance as preventable causes, as held by many recent articles [6,22,26,27,29,36,46,47]. Deaths occurring between 1996 and 1998 were coded using the International Classification of Diseases, 9^th^ edition (ICD-9); ICD-10 was used for deaths occurring between 1999 and 2007. For the first two causes, only deaths occurring before the age of 75 years were taken into account, following Nolte and McKee [5]. For the rest of causes, all deaths occurring were taken into account.

**Table 1:** Frequencies and percentages with regard to the overall mortality by sex, age, period and cause of death(*) in all the 33 cities studied

**Period**	**Sex**	**Age**	**Lung cancer**		**Cirrhosis**		**AIDS, HIV**		**Motor vehicle injuries**		**Suicide**		**Homicide**		**All preventable**		**All causes**	
			**n**	**%**	**n**	**%**	**n**	**%**	**n**	**%**	**n**	**%**	**n**	**%**	**n**	**%**	**n**	**%**
1996-2001	Men	0-44	744	2.9	866	3.4	4370	17.1	3209	12.5	1524	6.0	308	1.2	11021	43.1	25595	100
		45-64	8690	15.0	3232	5.6	798	1.4	1138	2.0	817	1.4	107	0.2	14782	25.5	58052	100
		>64	10427	4.4	2497	1.1	144	0.1	979	0.4	955	0.4	41	0.0	15043	6.3	237720	100
		Total	19861	6.2	6595	2.1	5312	1.7	5326	1.7	3296	1.0	456	0.1	40846	12.7	321367	100
	Women	0-44	274	2.5	209	1.9	1140	10.2	908	8.1	479	4.3	98	0.9	3108	27.9	11157	100
		45-64	1095	4.3	949	3.7	127	0.5	436	1.7	387	1.5	42	0.2	3036	11.9	25524	100
		>64	997	0.4	1458	0.6	30	0.0	693	0.3	493	0.2	44	0.0	3715	1.4	263798	100
		Total	2366	0.8	2616	0.9	1297	0.4	2037	0.7	1359	0.5	184	0.1	9859	3.3	300479	100
2002-2007	Men	0-44	656	3.1	702	3.3	1908	9.0	2486	11.7	1563	7.4	393	1.9	7708	36.4	21172	100
		45-64	9323	16.2	2988	5.2	876	1.5	900	1.6	984	1.7	148	0.3	15219	26.4	57622	100
		>64	9739	3.8	1962	0.8	136	0.1	810	0.3	1046	0.4	50	0.0	13743	5.4	256376	100
		Total	19718	5.9	5652	1.7	2920	0.9	4196	1.3	3593	1.1	591	0.2	36670	10.9	335170	100
	Women	0-44	316	3.2	195	2.0	561	5.7	582	5.9	535	5.4	133	1.3	2322	23.5	9896	100
		45-64	1958	7.5	803	3.1	122	0.5	276	1.1	525	2.0	62	0.2	3746	14.4	26095	100
		≥64	1230	0.4	966	0.3	24	0.0	547	0.2	492	0.2	55	0.0	3314	1.1	291098	100
		Total	3504	1.1	1964	0.6	707	0.2	1405	0.4	1552	0.5	250	0.1	9382	2.9	327089	100
1996-2007	Men	0-44	1400	3.0	1568	3.4	6278	13.4	5695	12.2	3087	6.6	701	1.5	18729	40.0	46767	100
		45-64	18013	15.6	6220	5.4	1674	1.4	2038	1.8	1801	1.6	255	0.2	30001	25.9	115674	100
		>64	20166	4.1	4459	0.9	280	0.1	1789	0.4	2001	0.4	91	0.0	28786	5.8	494096	100
		Total	39579	6.0	12247	1.9	8232	1.3	9522	1.5	6889	1.0	1047	0.2	77516	11.8	656537	100
	Women	0-44	590	2.8	404	1.9	1701	8.1	1490	7.1	1014	4.8	231	1.1	5430	25.8	21053	100
		45-64	3053	5.9	1752	3.4	249	0.5	712	1.4	912	1.8	104	0.2	6782	13.1	51619	100
		>64	2227	0.4	2424	0.4	54	0.0	1240	0.2	985	0.2	99	0.0	7029	1.3	554896	100
		Total	5870	0.9	4580	0.7	2004	0.3	3442	0.5	2911	0.5	434	0.1	19241	3.1	627568	100

Spain, 1996-2007.

(*)ICD codes and age: Lung cancer (ICD-9: 162; ICD-10: C33,C34; Age: 0–74), Cirrhosis (ICD-9: 571, 573.0; ICD-10: K70, K72.1, K73, K74, K76.1.9, Age: 0–74), AIDS and HIV (ICD-9: 279.1.5.6.8, 042, 795.8; ICD-10: B20-B24, R75; Age: all), Motor vehicle injuries (ICD-9: E810-E825; ICD-10: V02-V04, V09.0.2, V12-V14, V19.0.1.2.4.5.6, V20-V79, V80.3.4.5, V81.0.1, V82.0.1, V83-V86, V87-V88.0.1.2.3.4.5.6.7.8, V89.0.2; Age: all), Suicide (ICD-9: E950-E959; ICD-10: X60-X84; Age: all), Homicide (ICD-9: E960-E969; ICD-10: X85-Y09; Age: all).

To establish the socioeconomic status of each CT in each city the following indicators were used: Unemployment: percentage of people aged over 16 years out of work (unemployed people and first time job seekers), out of the total active population.

Education: percentage of people aged over 16 years who, according to the Spanish National Statistics Register figures, cannot read or write, can read and write but went to school for less than five years or for more than five years but without terminating primary studies, out of the total population aged 16 years and over.

Education in young people: percentage of persons aged between 16 and 29 years with low educational levels, out of the total population aged between 16 and 29 years.

Manual workers: percentage of persons aged 16 years employed in manual labour (services, agriculture, farming, fishing, crafts, specialised manufacture industry workers, construction, mining, installers and non-specialised workers) out of the total number of persons in employment aged 16 years or over.

Temporary workers: percentage of people aged 16 years or over employed in temporary jobs (part-time self-employed workers, temporary workers) out of the total of persons in employment aged 16 years or over.

These indicators had previously been used on the MEDEA project [48] and were used to build socioeconomic status (SES) variables in each city. Three levels were established: SES1 (the most privileged socioeconomic group), which includes all CTs in the city with values below the 25^th^ percentile for all five indicators; SES3 (the least privileged socioeconomic group), which represents all CTs with values above the 75^th^ percentile for all indicators; all other CTs were included in SES2 (intermediate socioeconomic level). This variable had previously been used to classify CTs by socioeconomic level and proved to be a good tool [22].

Frequencies and percentages (of total preventable deaths and death from all causes) were calculated for all causes studied and all preventable causes in each city. To study the trend in risk of death over time, the dates were classified into two time periods: 1996–2001 (P1) and 2002–2007 (P2) (In the cases of La Coruña, Ferrol, Lugo, Ourense, Pontevedra, Santiago and Vigo mortality figures were available from 1998 onwards, so for these cities the first period goes from 1998 to 2001). The age standardised mortality rates (ASR) and their 95% confidence intervals were calculated for all preventable cases, adjusted using the direct method and taking as standard the Spanish population in 2001 (centre of the period) by sex and age group, obtained from the NSI, for each city, in each period and by sex.

To analyse inequalities in mortality risks between the highest and lowest socioeconomic levels and over different periods, for each city and separating by sex, Poisson regression models with variable responses to the rate of death logarithm were adapted, using variables explaining SES (reference level:SES1), period (reference level: P1) and age, into three groups: younger than 45 years (reference level), 45 to 64 and older than 64 years. The age limit of 65 years was chosen as it is the cut-off age normally used in studies to define ‘old age’ [49]. Subjects aged under 65 were classified into two groups, depending on the lesser or greater prematurity of death. This model made it possible to estimate the Relative Risk (RR) of death and the corresponding 95% confidence intervals for each level of explicative SES variables, period and age group compared to the chosen reference level. We analysed possible interactions between SES and period, SES and age group and between age group and period. The existence of significant interaction between SES and period demonstrates the change between RR periods across socioeconomic levels. In order to control for overdispersion, which takes place when a certain distribution for the data is assumed and the variability of these data is higher than the one expected from the model assumed, quasi-likelihood models have been used. This kind of models enable a lack of precise likelihood for the answers and enable modelling on the basis of the linear predictor and the form assumed to represent the variance based on the average [50]. In the case of Poisson models, the variance function is supposed to differ only by a scale factor ϕ from the variance function in the corresponding likelihood model, i.e. Variance = ϕ Mean. In this way, the estimations of the parameters are equal. Nevertheless, the standard errors obtained by quasi-likelihood are the maximum likelihood estimators multiplied by ϕ ^½^. In order to control overdispersion in Poisson models, the parameter ϕ has been estimated. It has been tested whether it is statistically different from the unit. The estimator of ϕ takes the form D/df, where D is the deviance of the Poisson model adjusted, and df the degrees of freedom. If it is different from the unit, a quasi-Poisson model is adjusted, which is the appropriate model when there is overdispersion. This comparison and the estimation of all the models have been conducted by means of the R statistics package 2.12.2.

## Results

The populations and number of census tracts (CTs) in the cities analysed (Table 2) varied, according to 2001 figures, from a minimum of 75864 inhabitants and 57 sectors in Pontevedra to a maximum of 2874732 inhabitants and 2358 sectors in Madrid, with an mean population of 373283 inhabitants per city, with a mean of 297 sectors per city and a mean sector size of 1257 inhabitants.

**Table 2:** **Characteristics of the studied cities: population, number of census tracts (CTs) and percentage of sections in worse socioeconomic status (SES3)**

**City (Population 2001)**	**CTs**	**% CTs. in SES3**	Sex(*)	**Lung cancer**		**Cirrhosis**		**AIDS-VIH**		**Motor vehicle accidents**		**Suicide**		**Homicide**		**TOTAL preventable**	
				**n**	**%**	**n**	**%**	**n**	**%**	**n**	**%**	**n**	**%**	**n**	**%**	**n**	**%**
ALICANTE	215	9.3	M	921	48.4	301	15.8	199	10.5	259	13.6	191	10.0	32	1.7	1903	100
(283243)			W	144	28.9	131	26.3	53	10.6	81	16.3	77	15.5	12	2.4	498	100
ALMERIA	118	10.2	M	443	42.2	152	14.5	107	10.2	209	19.9	103	9.8	36	3.4	1050	100
(176709)			W	38	18.4	51	24.8	19	9.2	54	26.2	36	17.5	8	3.9	206	100
AVILES	72	8.3	M	352	54.0	109	16.7	40	6.1	77	11.8	66	10.1	8	1.2	652	100
(83553)			W	42	30.9	27	19.9	8	5.9	33	24.3	23	16.9	3	2.2	136	100
BARCELONA	1491	8.7	M	5761	56.5	1563	15.3	985	9.7	869	8.5	920	9.0	90	0.9	10188	100
(1505336)			W	900	31.4	813	28.3	262	9.1	383	13.3	450	15.7	62	2.2	2870	100
BILBAO	288	8.0	M	1356	50.8	458	17.2	310	11.6	268	10.0	255	9.6	23	0.9	2670	100
(349972)			W	244	31.5	181	23.4	110	14.2	107	13.8	117	15.1	16	2.1	775	100
CADIZ	111	11.7	M	522	49.5	237	22.5	133	12.6	94	8.9	66	6.3	3	0.3	1055	100
(133242)			W	46	17.7	122	46.9	29	11.2	25	9.6	34	13.1	4	1.5	260	100
CARTAGENA-LU	146	8.2	M	667	44.7	221	14.8	123	8.2	291	19.5	161	10.8	30	2.0	1493	100
(212952)			W	70	22.9	73	23.9	36	11.8	70	22.9	48	15.7	9	2.9	306	100
CASTELLON	95	5.3	M	481	48.0	118	11.8	94	9.4	194	19.4	107	10.7	8	0.8	1002	100
(146563)			W	59	27.1	39	17.9	14	6.4	54	24.8	46	21.1	6	2.8	218	100
CORDOBA	224	10.7	M	885	46.0	336	17.5	291	15.1	222	11.5	174	9.0	17	0.9	1925	100
(319692)			W	79	21.6	81	22.2	60	16.4	81	22.2	52	14.2	12	3.3	365	100
CORUNA	181	4.4	M	733	58.5	121	9.7	99	7.9	141	11.3	149	11.9	10	0.8	1253	100
(239434)			W	96	28.5	62	18.4	25	7.4	77	22.8	72	21.4	5	1.5	337	100
FERROL	69	7.2	M	274	55.6	82	16.6	36	7.3	48	9.7	49	9.9	4	0.8	493	100
(80347)			W	43	31.9	30	22.2	16	11.9	23	17.0	21	15.6	2	1.5	135	100
GIJON	191	5.2	M	1063	50.5	379	18.0	161	7.6	227	10.8	250	11.9	26	1.2	2106	100
(269270)			W	150	28.5	113	21.5	50	9.5	104	19.8	101	19.2	8	1.5	526	100
GRANADA	181	12.7	M	589	39.6	324	21.8	192	12.9	206	13.9	152	10.2	23	1.5	1486	100
(237720)			W	88	22.6	114	29.2	28	7.2	67	17.2	78	20.0	15	3.8	390	100
HUELVA	101	10.9	M	475	47.5	150	15.0	178	17.8	128	12.8	61	6.1	8	0.8	1000	100
(144369)			W	44	21.9	49	24.4	36	17.9	34	16.9	32	15.9	6	3.0	201	100
JAEN	76	11.8	M	250	37.8	200	30.3	40	6.1	73	11.0	88	13.3	10	1.5	661	100
(115917)			W	23	16.1	60	42.0	8	5.6	21	14.7	29	20.3	2	1.4	143	100
LAS PALMAS	263	9.1	M	981	47.5	415	20.1	178	8.6	237	11.5	224	10.8	31	1.5	2066	100
(364775)			W	178	36.8	93	19.2	42	8.7	74	15.3	86	17.8	11	2.3	484	100
LOGRONO	91	4.4	M	333	42.7	85	10.9	76	9.7	166	21.3	116	14.9	4	0.5	780	100
(131143)			W	39	20.2	27	14.0	14	7.3	68	35.2	40	20.7	5	2.6	193	100
LUGO	69	2.9	M	216	51.2	44	10.4	24	5.7	81	19.2	51	12.1	6	1.4	422	100
(88901)			W	29	20.7	24	17.1	5	3.6	49	35.0	28	20.0	5	3.6	140	100
MADRID	2358	7.5	M	9213	54.5	2504	14.8	2276	13.5	1643	9.7	969	5.7	312	1.8	16917	100
(2874732)			W	1593	37.5	960	22.6	498	11.7	733	17.2	362	8.5	104	2.4	4250	100
MALAGA	422	9.2	M	1708	46.0	653	17.6	448	12.1	479	12.9	359	9.7	67	1.8	3714	100
(546601)			W	202	24.9	238	29.3	85	10.5	107	13.2	168	20.7	11	1.4	811	100
MURCIA	295	3.7	M	960	44.4	384	17.7	118	5.5	461	21.3	207	9.6	34	1.6	2164	100
(398815)			W	103	26.1	85	21.5	24	6.1	97	24.6	71	18.0	15	3.8	395	100
OURENSE	79	3.8	M	275	51.8	53	10.0	40	7.5	97	18.3	61	11.5	5	0.9	531	100
(109051)			W	51	35.4	19	13.2	7	4.9	27	18.8	33	22.9	7	4.9	144	100
OVIEDO	173	8.7	M	720	51.4	235	16.8	106	7.6	160	11.4	163	11.6	16	1.1	1400	100
(201005)			W	118	33.7	54	15.4	23	6.6	77	22.0	71	20.3	7	2.0	350	100
PAMPLONA	122	4.1	M	562	52.7	97	9.1	79	7.4	173	16.2	146	13.7	9	0.8	1066	100
(173272)			W	105	35.4	33	11.1	30	10.1	72	24.2	53	17.8	4	1.3	297	100
PONTEVEDRA	57	5.3	M	191	50.7	36	9.5	38	10.1	73	19.4	38	10.1	1	0.3	377	100
(75864)			W	25	24.8	22	21.8	14	13.9	23	22.8	17	16.8	0	0.0	101	100
SAN SEBASTIAN	140	5.7	M	573	50.8	186	16.5	88	7.8	159	14.1	111	9.8	11	1.0	1128	100
(178377)			W	130	37.8	58	16.9	25	7.3	60	17.4	66	19.2	5	1.5	344	100
S. C. TENERIFE	157	6.4	M	606	49.4	227	18.5	130	10.6	129	10.5	124	10.1	11	0.9	1227	100
(214153)			W	115	42.8	58	21.6	26	9.7	35	13.0	30	11.2	5	1.9	269	100
SANTIAGO	73	4.1	M	242	54.6	48	10.8	23	5.2	86	19.4	42	9.5	2	0.5	443	100
(93381)			W	42	36.2	15	12.9	6	5.2	42	36.2	10	8.6	1	0.9	116	100
SEVILLA	510	12.7	M	2205	49.2	800	17.9	481	10.7	530	11.8	408	9.1	55	1.2	4479	100
(704305)			W	256	28.6	230	25.7	89	9.9	151	16.9	154	17.2	15	1.7	895	100
VALENCIA	533	8.5	M	2623	49.5	847	16.0	629	11.9	663	12.5	456	8.6	80	1.5	5298	100
(746612)			W	345	23.8	429	29.6	206	14.2	191	13.2	246	17.0	34	2.3	1451	100
VIGO	236	4.7	M	751	52.0	182	12.6	126	8.7	217	15.0	143	9.9	26	1.8	1445	100
(287282)			W	133	34.3	72	18.6	37	9.5	81	20.9	56	14.4	9	2.3	388	100
VITORIA	168	4.2	M	559	44.2	184	14.6	98	7.8	245	19.4	166	13.1	12	0.9	1264	100
216852)			W	81	24.9	60	18.5	36	11.1	91	28.0	51	15.7	6	1.8	325	100
ZARAGOZA	462	5.4	M	2089	54.1	516	13.4	286	7.4	617	16.0	313	8.1	37	1.0	3858	100
(614905)			W	259	28.1	157	17.0	83	9.0	250	27.1	153	16.6	20	2.2	922	100

(*) M: Men, W:Women.

Frequency and percentage of deaths (relative to the total preventable deaths) for each preventable cause, sex and city. Spain, 1996–2007.

The total number of preventable deaths for all cities in the period analysed was 96757. Of these, 77516 were men and 19241 were women, accounting for 11.8% and 3.1% of all deaths, respectively (Table 1). By periods and for all cities, there was a reduction in the number of preventable deaths in men, from 40846 in the 1996–2001 period to 36670 in the 2002–2007 period. This drop was attributable mainly to AIDS and HIV, road traffic accidents, cirrhosis and, to a lesser degree, lung cancer, while the number of suicides and homicides rose. There was also a reduction, albeit a smaller one, in the number of preventable deaths among women, from 9859 in the first period to 9382 in the second, with fewer deaths from AIDS, HIV, cirrhosis and road traffic accidents, with higher numbers of deaths from lung cancer, suicide and homicide.

Table 2 shows the frequencies and percentages of deaths (compared to total preventable deaths) per city, sex and specific cause. The most frequent cause for men, in all cities, was lung cancer, with a percentage out of total deaths from all causes varying from 37.8% in Jaén to 58.5% in Coruña. In the case of women, in 20 of the 33 cities analysed (60.6%) the most frequent cause of death was lung cancer, in 7 cities (21.2%) it was cirrhosis, and in 6 (18.2%) it was road traffic accidents.

Table 3 shows the age standardized rates (ASRs) for all preventable deaths studied for each city, sex and period. In the case of men, there is an average decrease of 15.7%, with a decrease of all the rates adjusted in every city. Women showed a mean reduction of 11.3%, although some cities such as Avilés, Lugo, Pamplona and Pontevedra showed slight increases in the second period.

**Table 3:** **Age standardized mortality rates (95%CI) for the 33 cities studied, by sex and period**

**CITY**	**SEX**	**1996-2001**	**2002-2007**	**1996-2007**
ALICANTE	Men	134.1 (125.9-142.3)	99.3 (92.7-105.9)	115.5 (110.3-120.7)
	Women	29.1 (25.4-32.7)	26.1 (22.8-29.3)	27.5 (25.1-29.9)
ALMERIA	Men	130.2 (119.1-141.3)	111.1 (101.3-120.8)	120.1 (112.8-127.5)
	Women	21.9 (17.6-26.2)	19.9 (16.0-23.8)	20.9 (18.0-23.8)
AVILES	Men	131.3 (117.3-145.3)	114.4 (101.5-127.3)	122.5 (113.0-131.9)
	Women	22.8 (17.0-28.5)	25.3 (19.5-31.0)	24.2 (20.1-28.3)
BARCELONA	Men	119.2 (116.1-122.4)	93.9 (91.2-96.6)	105.9 (103.9-108.0)
	Women	28.7 (27.2-30.1)	24.3 (23.0-25.6)	26.4 (25.4-27.4)
BILBAO	Men	132.9 (126.0-139.7)	108.1 (101.9-114.2)	120.7 (116.1-125.3)
	Women	33.7 (30.4-37.0)	30.4 (27.3-33.6)	32.1 (29.8-34.4)
CÁDIZ	Men	152.6 (140.0-165.3)	121.9 (111.0-132.9)	137.1 (128.7-145.4)
	Women	35.5 (29.8-41.3)	25.5 (20.7-30.3)	30.5 (26.7-34.2)
CARTAGENA	Men	145.9 (135.3-156.4)	127.4 (118.2-136.6)	135.6 (128.7-142.5)
	Women	29.6 (25.1-34.2)	23.6 (19.7-27.5)	26.6 (23.6-29.6)
CASTELLÓN	Men	135.2 (123.6-146.8)	106.3 (96.7-115.9)	118.7 (111.3-126.1)
	Women	28.4 (23.2-33.5)	21.5 (17.3-25.7)	24.7 (21.4-28.0)
CORDOBA	Men	132.6 (124.5-140.8)	103.0 (96.3-109.8)	117.0 (111.7-122.2)
	Women	21.7 (18.6-24.8)	18.1 (15.4-20.8)	19.7 (17.7-21.7)
CORUÑA	Men	111.7 (102.1-121.4)	96.5 (89.4-103.5)	102.3 (96.6-107.9)
	Women	26.1 (21.8-30.4)	22.4 (19.2-25.5)	23.9 (21.4-26.5)
FERROL	Men	123.7 (106.6-140.9)	116.1 (102.7-129.6)	119.8 (109.2-130.4)
	Women	29.7 (21.6-37.7)	28.0 (21.8-34.2)	28.8 (23.9-33.7)
GIJÓN	Men	132.9 (125.1-140.8)	109.9 (103.0-116.8)	121.2 (116.0-126.4)
	Women	29.8 (26.1-33.4)	27.0 (23.6-30.3)	28.3 (25.8-30.7)
GRANADA	Men	128.5 (119.6-137.4)	103.4 (95.6-111.2)	115.3 (109.4-121.2)
	Women	25.1 (21.5-28.7)	25.4 (21.9-29.0)	25.2 (22.7-27.7)
HUELVA	Men	150.9 (137.7-164.1)	125.5 (114.2-136.8)	136.7 (128.2-145.3)
	Women	25.2 (20.2-30.3)	23.3 (18.7-27.8)	24.3 (20.9-27.7)
JAÉN	Men	126.3 (112.5-140.2)	112.6 (100.5-124.8)	118.6 (109.5-127.6)
	Women	23.6 (17.9-29.3)	22.5 (17.4-27.6)	23.1 (19.3-26.9)
LAS PALMAS	Men	121.4 (114.2-128.6)	89.9 (84.1-95.8)	104.9 (100.3-109.5)
	Women	25.3 (22.1-28.5)	21.3 (18.5-24.0)	23.1 (21.0-25.2)
LOGROÑO	Men	114.5 (103.4-125.5)	93.4 (83.8-103.0)	103.3 (96.0-110.5)
	Women	26.9 (21.7-32.1)	20.3 (16.1-24.6)	23.6 (20.2-26.9)
LUGO	Men	103.9 (88.4-119.5)	92.5 (81.0-104.0)	96.6 (87.4-105.9)
	Women	27.2 (19.8-34.6)	28.8 (22.7-34.8)	28.1 (23.4-32.8)
MADRID	Men	107.8 (105.6-110.0)	85.2 (83.2-87.1)	95.8 (94.3-97.2)
	Women	22.4 (21.5-23.4)	19.6 (18.7-20.4)	20.9 (20.2-21.5)
MÁLAGA	Men	144.4 (137.9-151.0)	122.4 (116.7-128.1)	132.5 (128.2-136.8)
	Women	27.2 (24.6-29.9)	24.2 (21.8-26.6)	25.7 (23.9-27.4)
MURCIA	Men	119.6 (112.4-126.8)	104.6 (98.3-110.9)	111.2 (106.5-115.9)
	Women	20.6 (17.7-23.4)	17.3 (14.9-19.8)	18.8 (16.9-20.6)
OURENSE	Men	110.5 (95.9-125.1)	93.9 (83.3-104.4)	100.1 (91.5-108.7)
	Women	23.8 (17.4-30.1)	23.6 (18.7-28.6)	23.7 (19.8-27.6)
OVIEDO	Men	124.8 (115.7-133.9)	107.6 (99.4-115.8)	115.9 (109.8-122.0)
	Women	27.6 (23.6-31.6)	22.3 (18.8-25.7)	24.8 (22.2-27.4)
PAMPLONA	Men	111.9 (102.4-121.3)	95.1 (86.8-103.3)	103.0 (96.8-109.2)
	Women	25.0 (20.8-29.3)	27.4 (23.1-31.7)	26.1 (23.1-29.1)
PONTEVEDRA	Men	120.3 (101.5-139.0)	100.7 (87.3-114.2)	108.0 (97.0-118.9)
	Women	23.9 (16.0-31.7)	26.3 (19.9-32.8)	25.2 (20.3-30.2)
SAN SEBASTIÁN	Men	114.2 (104.9-123.4)	96.4 (88.2-104.6)	105.5 (99.3-111.7)
	Women	31.2 (26.7-35.7)	24.1 (20.3-27.9)	27.7 (24.8-30.7)
SANTA CRUZ de TENERIFE	Men	113.3 (104.2-122.3)	101.0 (92.9-109.0)	106.9 (100.9-112.9)
	Women	21.0 (17.3-24.6)	20.5 (17.1-24.0)	20.8 (18.3-23.3)
SANTIAGO	Men	101.4 (86.3-116.5)	99.9 (87.9-111.9)	100.7 (91.3-110.1)
	Women	29.1 (21.5-36.8)	19.4 (14.5-24.3)	23.2 (19.0-27.5)
SEVILLA	Men	133.1 (127.7-138.6)	109.7 (105.0-114.4)	120.9 (117.4-124.5)
	Women	21.8 (19.8-23.8)	20.2 (18.3-22.1)	21.0 (19.6-22.4)
VALENCIA	Men	135.0 (130.1-140.0)	107.3 (103.0-111.5)	120.4 (117.1-123.6)
	Women	33.5 (31.1-35.8)	25.6 (23.6-27.6)	29.4 (27.9-31.0)
VIGO	Men	107.1 (98.2-115.9)	102.3 (95.5-109.1)	104.4 (99.0-109.8)
	Women	25.8 (21.7-30.0)	24.9 (21.7-28.1)	25.2 (22.7-27.7)
VITORIA	Men	105.2 (97.1-113.3)	87.3 (80.3-94.3)	95.8 (90.5-101.1)
	Women	30.8 (26.4-35.2)	18.6 (15.3-21.8)	24.4 (21.7-27.1)
ZARAGOZA	Men	110.8 (105.9-115.6)	95.8 (91.3-100.2)	103.1 (99.9-106.4)
	Women	23.2 (21.1-25.3)	22.6 (20.5-24.6)	22.9 (21.4-24.4)

Spain, 1996-2007.

**Figure 2:**
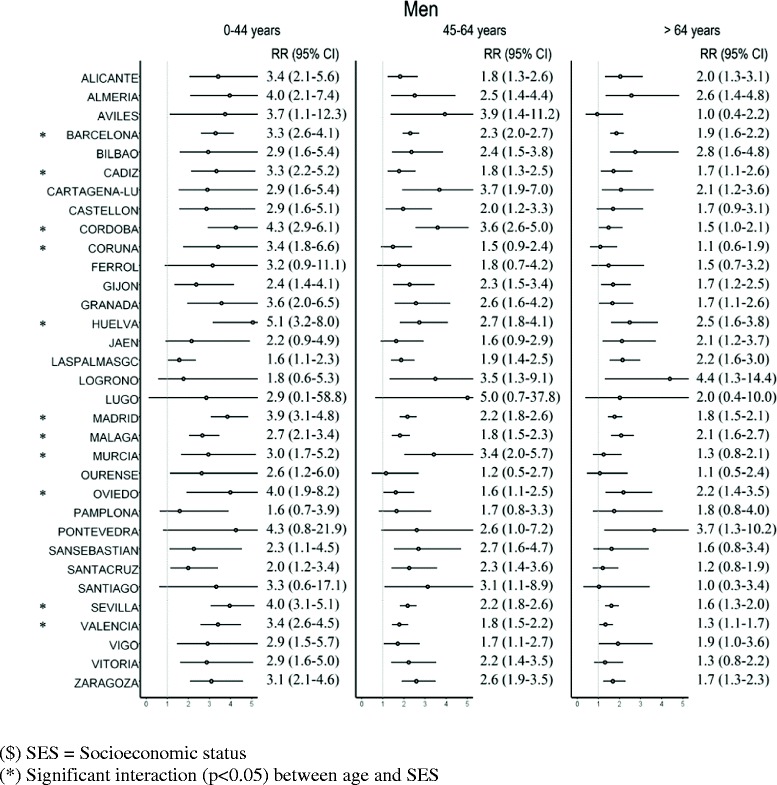
**Relative Risk (RR) of death and 95% confidence interval (95% CI) in SES3 vs. SES1** ^**$**^ **for men in each city, by age group.**

**Figure 3:**
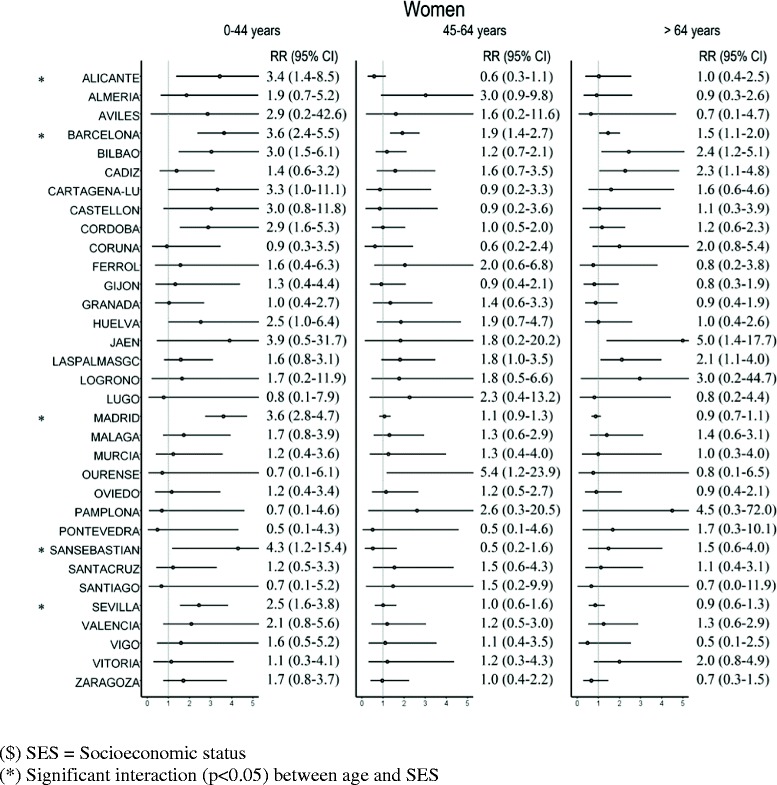
**Relative Risk (RR) of death and 95% confidence interval (95% CI) in SES3 vs. SES1** ^**$**^ **for women in each city, by age group.**

Figures 2 and 3 show the RRs of death between the least privileged and most privileged levels (SES3 and SES1, respectively) of the SES variables estimated using Poisson regression. These relative risks show the excess risk of death at the lowest level (SES3) compared to the highest level (SES1). The estimated RRs are presented by age groups, as significant interaction between age group and SES was detected in several cases. Nevertheless, no significant interaction was detected between the period under analysis and SES, there being accordingly no evidence that RRs among SES levels vary between periods in any city, in men or in women.

In men (Figure 2) the RR of death in SES3 compared to SES1 was higher than 1 in all cities and at all ages, significantly so (p < 0.05) in 26 cities in the 0–44 age group, in 27 cities in the 45–64 age group, and in 21 cities in the group aged over 64 years. Significant interaction was detected between age group and SES in 11 of the 33 cities analysed, while in the rest the SES effect was constant over the three age groups. In general, the interaction detected translates into higher RRs among SES levels for the youngest groups. This is the case, for example, of Madrid, with RRs of 3.9 in the 0–44 age group, 2.2 in the 45–64 age group, 2.2 in the 45–64 age group and 1.8 in the group aged 65 or over.

In the case of women (Figure 3), the estimated RR were higher than 1 in 27 cities in the 0–44 age group (significantly so in 9 cities), in 26 cities in the 45–64 age group (significant in 3 cities) and in 20 cities in the group aged 64 years and older (significant in 5 cities). There was significant interaction between SES and age group in 5 of the 33 cities studied; in the rest the SES effect was constant over the three age groups. The interaction effect detected in these cities was due, as in men, to significant excesses in risk among younger women.

Table 4 shows the median and mean calculated using the relative risks of the 33 cities when SES3 and SES1 are compared. It shows that, on average, the RRs for the SES variable decreased with age, being highest in the 0–44 years age group.

**Table 4:** **Median, 10% truncated mean and standard deviation for the Relative Risk when comparing SES3 vs. SES 1** ^**$**^ **in the studied cities, by age group, period and sex**

**Age group**	**Period**	**Men**				**Women**			
		**Median**	n(*)	**Mean**	**SD**	**Median**	n(*)	**Mean**	**SD**
0-44 years	1996-2001	3.3	27	3.3	1.1	2.1	28	2.2	1.6
	2002-2007	3.0	27	3.0	0.7	1.9	28	2.4	1.5
45-64 years	1996-2001	2.1	27	2.2	0.6	1.3	28	1.6	1.2
	2002-2007	2.3	28	2.4	0.8	1.2	28	1.3	0.5
>64 years	1996-2001	1.8	27	1.9	0.6	1.0	27	1.4	0.8
	2002-2007	1.7	29	1.7	0.5	1.2	27	1.5	0.9

($) SES = Socioeconomic status.

(*) Number of cities.

## Discussion

This is the first time that preventable avoidable mortality has been analyzed in such a high number of Spanish cities. Using data from thirty-three major Spanish cities, basic socioeconomic indicators of the educational and working environment have been used in this study to examine socioeconomic inequalities in preventable avoidable mortality and thus to detect urban areas to investigate possible problems in the chain of primary prevention, both in the promotion and protection of health and in health education, or over which should address specific health policies. The existence of preventable mortality inequalities should be an indicator of differences in health policy outcomes between different socioeconomic groups. The results of this study show that preventable avoidable mortality made a significant contribution to general mortality (around 7.5%, higher among men), having clearly decreased over time in men (12.7 in 1996–2001 and 10.9 in 2002–2007), though not so clearly among women (3.3% in 1996–2001 and 2.9% in 2002–2007). In the thirty-three cities studied, it has been observed in men, with great consistency, that the risks of death are higher in areas of greater deprivation, and that these excesses have not modified over time. The result in women was different and differences in mortality risks by socioeconomic level could not be established in many cities.

Preventable deaths as a percentage of general mortality dropped between the first and second periods. Men contributed more heavily to the reduction, which was a consequence of the drop in mortality from AIDS and HIV, road traffic injuries, cirrhosis and other liver diseases and, to a lesser degree, lung cancer. For men there was a drop in ASRs in all cities. The effect was different in women, who experienced an increase in the percentage of avoidable deaths between the first and second period, due to lung cancer, suicide and homicide. Consequently, there was greater variability in mortality risk trends, and ASRs increased between the first and second periods in several cities.

The general downward tendency coincides with other studies on avoidable mortality [29,30]. Grabauskas et al. described a growing tendency in avoidable mortality in Lithuania [33]. By causes, in Spain, Dalmau-Bueno et al. found a decrease in deaths due cirrhosis between 1992 and 2004 in Barcelona, in both men and women [23]. Other studies have described a reduction in AIDS and HIV mortality, particularly as a consequence of Highly Active Antiretroviral Therapy (HAART), among them Borrell et al. in Barcelona [19] and Regidor et al. in the Madrid region [24]. With regard to lung cancer, the general tendency in European countries points towards a reduction among men (particularly young men) and an increase among women [51]. In Spain, the results are similar, rising among women and falling among men [32].

The socioeconomic inequalities found in this study for all deaths from preventable causes confirm the others. Thus, Gotsens et al. found socioeconomic inequalities in 15 European cities for death from injuries (road traffic injuries, suicide, homicide, other external causes) between 2000 and 2008 [26]. Socioeconomic inequalities were also found in AIDS and HIV mortality in cities such as Barcelona [20], in mortality from cirrhosis in Zaragoza [25] or inequalities in educational level in mortality from AIDS, cirrhosis and accident and suicide injuries in the Madrid region [18], and for the lung cancer, cirrhosis, motor traffic accident injuries and AIDS in Castellón, Valencia and Alicante [22].

In this study we found no changes in time in the effects of socioeconomic inequalities on mortality, as the effects of the interaction between SES and period were not significant in any city. Thus we may interpret that the inequalities remained constant in both periods. Considering the 33 cities studied, in the case of men the median RR between SES3 and SES1 did not differ between the two periods for any age group, varying from 1.7 and 1.8, in the group aged over 65 years, in the first and second periods, respectively, and 3.3 (period 1) and 3.0 (period 2) in the group aged under 45 years. These findings consistently show the existence of generalised socioeconomic inequalities for all preventable causes analysed for men. In the case of women, the median for the three RRs is roughly 1 for the group aged over 65 years and, in the group aged under 45, 2.1 for the first period and 1.9 for the second one. Although the results are not significant in many cities and age groups, the estimated RRs are mainly higher than 1, indicating inequality between SES levels, though not quite so pronounced as in men. The lack of statistical significance may be due to the lower number of deaths among women in comparison with men. The results obtained in the group aged over 65 years are in the same range as those presented by Huisman et al. [49], who reviewed socioeconomic inequalities in mortality in old age in the World Health Organization Europe Region and found that RR are rarely higher than 2.0.

Some studies in other countries point either to an increase in socioeconomic inequalities in preventable causes [36,52] or to their decrease [13]. In the case of the causes analysed here, results in trends in inequality vary. In a study conducted in Barcelona using figures from the 1992–2003 period, Borrell et al. [21] found that inequalities in mortality by educational level did not change substantially over time. There are studies which point to an increase in socioeconomic inequalities due to cirrhosis [34,53] or towards their remaining the same or increasing in certain age groups [23]. In the case of AIDS and HIV several studies show that socioeconomic inequalities have been maintained over time [19,20]. In the case of suicide, studies conducted in other countries have found that socioeconomic inequalities either remained steady [52,54] or increased over time [55]. In a study conducted on men in 26 Spanish cities, Gotsens et al. [27] found that socioeconomic inequalities in mortality from injuries (including drug overdose, road traffic injuries and suicide) did not change between the 1996–2001 and 2002–2007 periods. The results of this study are similar to those observed in a study conducted in Valencia, Castellón and Alicante analysing trends in socioeconomic inequalities in mortality from preventable causes (lung cancer, cirrhosis, traffic accidents injuries and AIDS) and which found RRs similar to those of this study, presenting a similar differential effect between men and women (higher RRs in men).

We believe special attention should be paid to age group inequalities, as this study shows that it is the under 45 years group where the greatest inequality occurs. In all cities where significant interaction was detected between SES and age (14 cities for men and 5 for women), it was in the under 45 years age group where the highest RR was estimated. This result offers clear guidelines for interventions aimed at reducing inequalities.

In a study conducted on 11 Spanish cities [56], varying patterns in relation between size of city and magnitude of socioeconomic inequalities in mortality were found, particularly a certain link to lung cancer and cirrhosis in men. Looking at the population characteristics of the cities analysed we can see that the geographical distribution of cities according to the percentage of CTs at the most deprived level (SES3) shows a certain grouping. Thus, the percentage for Lugo, Murcia, Ourense, Pamplona, Santiago, Vitoria, Logroño, Coruña, Vigo, Gijón, Castellón, Pontevedra, Zaragoza, San Sebastián, Santa Cruz de Tenerife and Ferrol is below the median, while the other cities – Madrid, Bilbao, Cartagena-La Unión, Avilés, Valencia, Oviedo, Barcelona, Las Palmas, Málaga, Alicante, Almería, Córdoba, Huelva, Cádiz, Jaén, Granada and Sevilla, are above it. We could say that the first group are clearly in the northern part of Spain (except Murcia and Castellón), while the rest are in the southern and Mediterranean parts (except Avilés, Oviedo and Bilbao). This result leads to the conclusion that higher proportions of deprived populations, according to the calculated indicator, lived in cities in the southern and Mediterranean regions of Spain. However, when we inspected the relationship between population size and percentage of CTs in level SES3 with socioeconomic inequalities (RRs of SES3 vs. SES1), low or moderate Spearman correlations were obtained (below 0.30 and non-significant), showing a weak relationship between inequalities and city size and percentage of deprived CTs.

This study has its limitations. First of all, we have to take into account that it is an ecological study, with the constraints inherent to this type of study. Thus, it does not allow the proof of a causal association. The association found between SES and mortality using CTs may not be applicable at an individual level (i.e. ecological fallacy) and the ecological associations found may reflect both the effect of individual socioeconomic level and the contextual effect of the area. Regarding the causes analysed, other lists could have been used. The causes were chosen on the basis of comparability with other studies and it should be taken into account that exposure to risk factors for some of the causes analysed may have occurred in places other than the place of residence, such as in the work place, as the persons most exposed may live in very deprived neighbourhoods. Nevertheless, an analysis using small areas makes it possible to chase down and identify populations at risk, although some of the exposure may occur outside of them. Another constraint may arise from the use of different mortality classifications throughout the study period. In 1999, there was a switchover from ICD-9 to ICD-10. Two studies conducted in Spain concluded that the introduction of ICD-10 caused no important changes to the causes analysed here [57,58]. The classification of CTs into SES was performed using accumulated data from the 2001 census and remained the same throughout the study period, which was relatively short. Regarding SES classifications used in this study, we should mention that the relative risks estimated between the most favoured and least favoured categories show, for each city, the relative risk between the worst and the best population group in all indicators used. Thus, the interpretation of these relative risks differs from that obtained using other classifications based on percentiles or the continuous value of socioeconomic indicators composed from originals, and reports the level of extreme inequalities between categories which could be identified as maximum and minimum deprivation. This classification makes it possible to identify the most deprived areas, which require greater surveillance and attention, and the consistency of the results obtained using this classification is worth noting. Another aspect to be taken into account is that the term mortality, avoidable or not, takes only deaths, but not other health outcomes, into consideration. This gives an limited view of overall health outcomes, such as suicides, by not providing information about suicide attempts instead of deaths, or that it is not the best indicator of the efficacy of preventive measures, such as the reduction in accident injuries or improvements in quality of life as outcomes other than death.

The mortality analysed in this study may be reduced by means of well-designed health interventions and policies aimed at preventing disease and disability. Several measures established by Spanish governments since the beginning of this century, for example in road safety, established as a priority in 2004, or campaigns aimed at reducing tobacco use, which made it possible to introduce the smoking ban, may be effective in reducing mortality, but transferring the effects of these measures to the reduction of socioeconomic inequalities in mortality requires investments in public health activities to promote health and prevent disease. These activities include efforts in monitoring the state of health of the community, investigating the areas at most risk, educating the population regarding health risks and prevention strategies, intensifying health promotion initiatives, and reinforcing and adapting laws and regulations. In this line, primary healthcare may play an important role in contributing to reducing health inequalities. Hernández et al. [59] proposes recommendations made by the Spanish Commission for the Reduction of Health Inequalities, for putting actions of this type into effect.

The period studied here falls within a period of economic boom in the country, ending in 2007, when the current world economic crisis, which having serious effects in Spain, commenced. Accordingly, this work aims to serve as a point of reference for future studies which evaluate trends in inequalities in preventable mortality in later periods.

## Conclusions

This study shows that preventable mortality analysed decreased between the 1996–2001 and 2002–2007 periods, more markedly in men than in women, and that there were socioeconomic inequalities in mortality in most cities analysed, particularly among the youngest population, associating a higher risk of death with higher levels of deprivation. Moreover, inequalities emained over the two periods analysed. This type of study makes it possible to identify those areas where excess preventable mortality is associated with more deprived zones. It is in these deprived zones where actions to reduce and monitor health inequalities should be put into place. Primary healthcare may play an important role in this process.

## Abbreviations

CT: Census tract
NSI: National statistics institute
AIDS and HIV: Acquired immune deficiency syndrome and human immunodeficiency virus infection
ICD-9: International classification of diseases, 9^th^ version
ICD-10: International classification of diseases, 10^th^ version
SES: Socioeconomic status
SES1: Most privileged socioeconomic status
SES2: Intermediate socioeconomic status
SES3: Least privileged socioeconomic status
P1: Time period 1996–2001
P2: Time period 2002–2007
ASR: Age standardized rate
RR: Relative risk
IHD: Ischaemic heart disease
